# Supplementary material for: TAF1 plays a critical role in AML1-ETO driven leukemogenesis
Source: Nat Commun. 2019 Oct 29;10:4925. doi: 10.1038/s41467-019-12735-z (PMC6820555; doi:10.1038/s41467-019-12735-z)
Supplement: Supplementary file 1 — Supplementary Information [file 41467_2019_12735_MOESM1_ESM.pdf]

TAF1 plays a critical role in AML1-ETO driven leukemogenesis

Ye Xu et al.

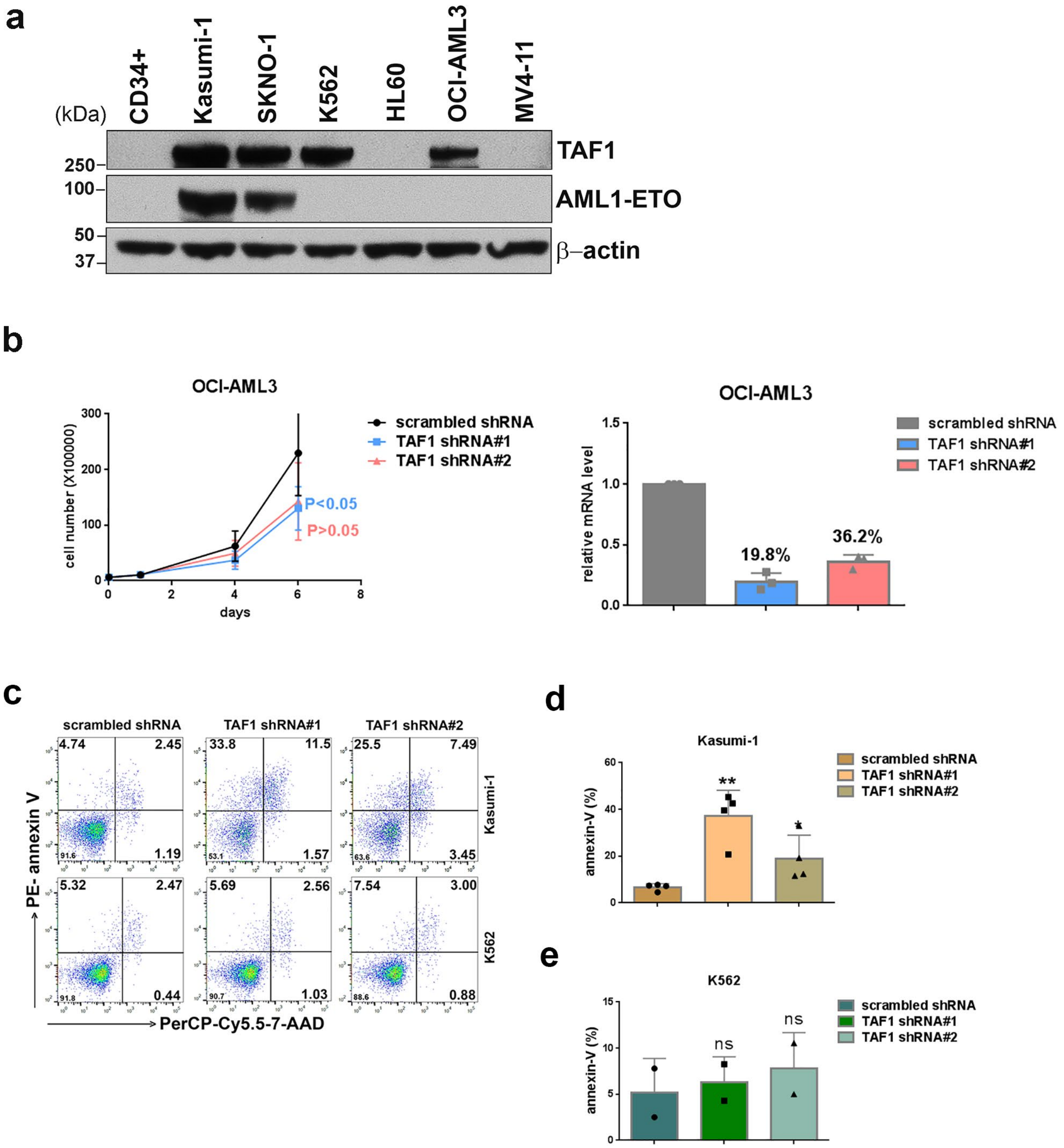

Supplementary Figure 1. The knockdown of TAF1 has minor effect on the growth of OCI-AML3 cells and induces apoptosis in Kasumi-1 cells. (a) The protein level of TAF1 in CD34+ HSPCs and AML cell lines. (b) TAF1 KD has minimal effect on the growth of OCI-AML3 cells. OCI-AML3 cells were infected with scrambled shRNA or two TAF1 directed shRNAs. The numbers of cells were counted at the indicated times (left panel). The cell numbers between cells infected with scrambled shRNA and TAF1 shRNAs at day 6 were compared using Student t test. p values are displayed. The levels of TAF1 expression in each type of cells are shown in the right panel and the percentage of the remaining level of TAF1 in each type of cells after TAF1 KD are shown on each columns . (c-e) Lack of TAF1 induces apoptosis in Kasumi-1 cells, but not in K562 cells. Kasumi-1 cells and K562 cells were transduced with scrambled shRNA or TAF1 directed shRNAs for 4 days and then subjected to apoptotic assays. Representative flow cytometry pictures are shown in (c). (d-e) The percentage of apoptotic cells in Kasumi-1 cells (d) or K562 cells (e) transduced with scrambled shRNA or TAF1 shRNAs are shown in bar graphs. The comparison of the percentage of apoptotic cells was performed between cells transduced with scrambled shRNA and each TAF1 shRNA by Student t test. * indicates $p < 0.05$, ** indicates $p < 0.01$ and "ns" indicates $p > 0.05$. (a-e) All experiments were repeated independently at least two times, (b) n =3, (d) n = 4, (e) n = 2. All error bars represent mean ± SD.

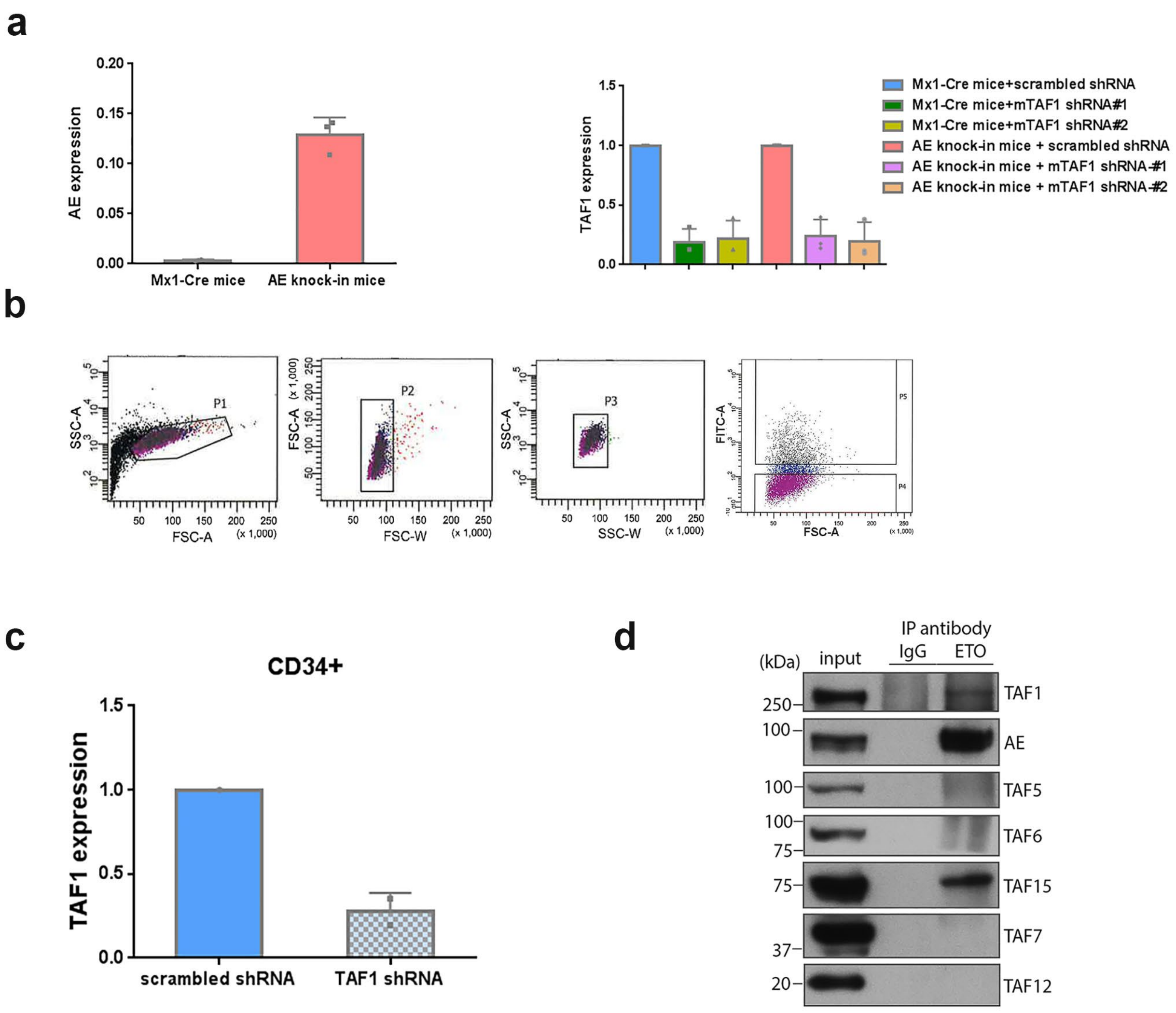

Supplementary Figure 2. The expression of TAF1 and AE in bone marrow and CD34+ cells. (a) AE was expressed in AE knock-in mice (left panel) and TAF1 was knocked down using mouse TAF1 shRNAs in bone marrow cells isolated from Mx1-Cre mice and AE knock-in mice (right panel). Experiments were repeated independently three times and error bars represent mean ± SD. (b) Gating strategy for GFP+ AE-expressing CD34+ cells. CD34+ cells with normal or reduced level of TAF1 were infected with MigR1 or MigR1-AE viruses for 2 days and then GFP+ MigR1 cells or MigR1-AE expressing cells were sorted by flow cytometry. Non infected CD34+ cells were used as negative control. (c) TAF1 depletion levels in human CD34+ CB cells. Experiments were repeated independently two times and error bars represent mean ± SD. (d) Co-immunoprecipitation assays were performed using an anti-ETO antibody in Kasumi-1 cells.

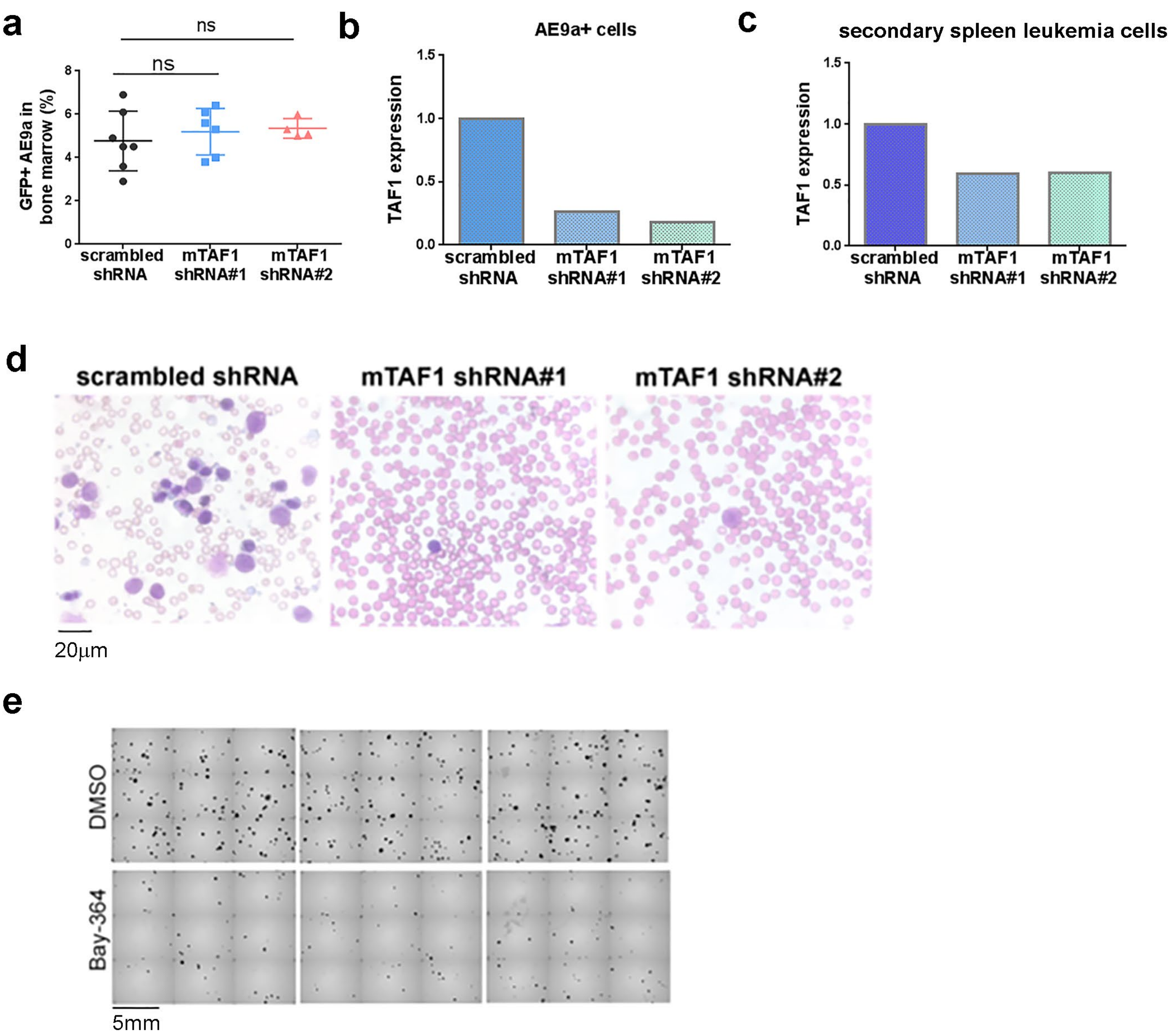

Supplementary Figure 3. TAF1 is critical for leukemia development. (a) Depletion of TAF1 does not affect the homing of AE9a+ luciferase+ cells to the bone marrow of transplanted recipient mice. Bone marrow cells were collected 16 hours after transplantation. The percentage of GFP+ AE9a+ cells in peripheral blood of mice injected with AE9a+ cells transduced with scrambled shRNA was compared with that in the mice injected with AE9a+ cells transduced with TAF1 shRNAs. p value was determined by Student t test. ns indicates $p > 0.05$. (b-c) The mRNA level of TAF1 in AE9a+ luciferase+ cells (b) or secondary spleen leukemia cells (c) after transduction of scrambled shRNA or mouse TAF1 shRNAs. (d) Depletion of TAF1 abrogates the growth of leukemia cells in peripheral blood. Representative images show the H&E staining of peripheral blood from mice transplanted with secondary spleen leukemia cells infected with scrambled shRNA or mouse TAF1 shRNAs for 7 weeks. (e) Bay-364 inhibits the colony formation of AE9a+ cells. AE9a+ cells were treated with vehicle or 10uM Bay-364 for 2 days and same numbers of cells were plated on methylcellulose-based medium containing vehicle or 3 uM Bay-364 for 7 days. The 6-well plates were scanned using STEMvison software. The experiments were repeated independently three times. Three representative areas in each treatment group are shown.

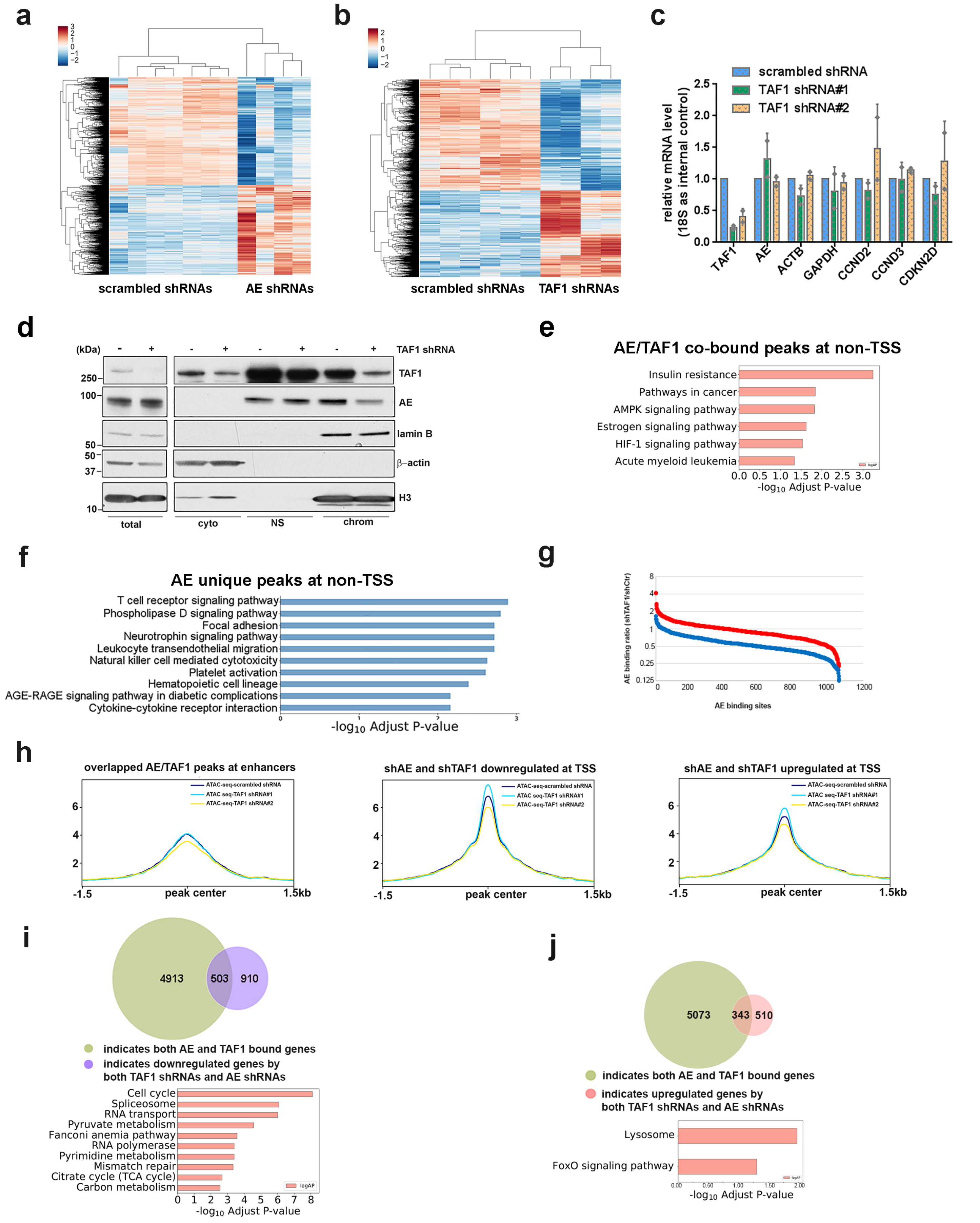
a
scrambled shRNAs
AE shRNAs
b
scrambled shRNAs
TAF1 shRNAs
c
scrambled shRNA
TAF1 shRNA#1
TAF1 shRNA#2
relative mRNA level (18S as internal control)
TAF1
AE
ACTB
GAPDH
CCND2
CCND3
CDKN2D
d
(kDa)
TAF1 shRNA
TAF1
AE
lamin B
β-actin
H3
total
cyto
NS
chrom
e
AE/TAF1 co-bound peaks at non-TSS
Insulin resistance
Pathways in cancer
AMPK signaling pathway
Estrogen signaling pathway
HIF-1 signaling pathway
Acute myeloid leukemia
-log10 Adjust P-value
f
AE unique peaks at non-TSS
T cell receptor signaling pathway
Phospholipase D signaling pathway
Focal adhesion
Neurotrophin signaling pathway
Leukocyte transendothelial migration
Natural killer cell mediated cytotoxicity
Platelet activation
Hematopoietic cell lineage
AGE-RAGE signaling pathway in diabetic complications
Cytokine-cytokine receptor interaction
-log10 Adjust P-value
g
AE binding ratio (shTAF1/shCtr)
AE binding sites
h
overlapped AE/TAF1 peaks at enhancers
shAE and shTAF1 downregulated at TSS
shAE and shTAF1 upregulated at TSS
ATAC-seq-scrambled shRNA
ATAC seq-TAF1 shRNA#1
ATAC-seq-TAF1 shRNA#2
-1.5
peak center
1.5kb
i
4913
503
910
indicates both AE and TAF1 bound genes
indicates downregulated genes by both TAF1 shRNAs and AE shRNAs
Cell cycle
Spliceosome
RNA transport
Pyruvate metabolism
Fanconi anemia pathway
RNA polymerase
Pyrimidine metabolism
Mismatch repair
Citrate cycle (TCA cycle)
Carbon metabolism
-log10 Adjust P-value
j
5073
343
510
indicates both AE and TAF1 bound genes
indicates upregulated genes by both TAF1 shRNAs and AE shRNAs
Lysosome
FoxO signaling pathway
-log10 Adjust P-value

Supplementary Figure 4. The KD of AE or TAF1 changes the expression of a subset of AE regulated genes. (a-b) Heat maps of differentially expressed gene in Kasumi-1 cells infected with AE shRNAs (a) or TAF1 shRNAs (b) compared to those infected with scrambled shRNA. Heat maps were generated from variance stabilized counts of genes differentially expressed with a BH FDR $q < 0.05$ and a fold change +/- 1.5 vs control. (c) The influence of TAF1 knockdown on the expression of housekeeping genes and cell cycle regulatory genes in Kasumi-1 cells. Kasumi-1 cells were transduced with scrambled shRNA or TAF1 shRNAs for 5 days. mRNA levels of individual genes were standardized by the level of 18S rRNA. (d) TAF1 KD reduces the association of AE with chromatin 3 days after TAF1 KD. "total" indicates the whole cell extract; "cyto" indicates the cytoplasm fraction; "NS" indicates the nuclear soluble fraction; "chrom" indicates the chromatin fraction. (e-f) KEGG analysis was performed for genes adjacent to AE/TAF1 overlapping peaks at non-TSS (e) or AE unique peaks at non-TSS (f) (g) AE binding signals are compared at 1,061 AE binding sites which are occupied by TAF1 between cells with TAF1 KD and cells with normal TAF1 level. The ratios (shTAF1/shCtr) displayed on the y-axis are ranked from high to low at each site as shown on the x-axis. Two biological replicates are shown in different colors. (h) TAF1 KD does not change the global chromatin accessibility at AE and TAF1 overlapped peaks at enhancer regions (left), nor at the TSS regions of downregulated (middle) and upregulated (right) genes by both AE and TAF1 KD. (i-j) Venn diagrams illustrate the genes adjacent to co-localized TAF1 and AE peaks overlapping with genes downregulated (i) or upregulated (j) ($q < 0.05$) by both TAF1 and AE shRNAs. KEGG gene sets were performed on overlapping genes.

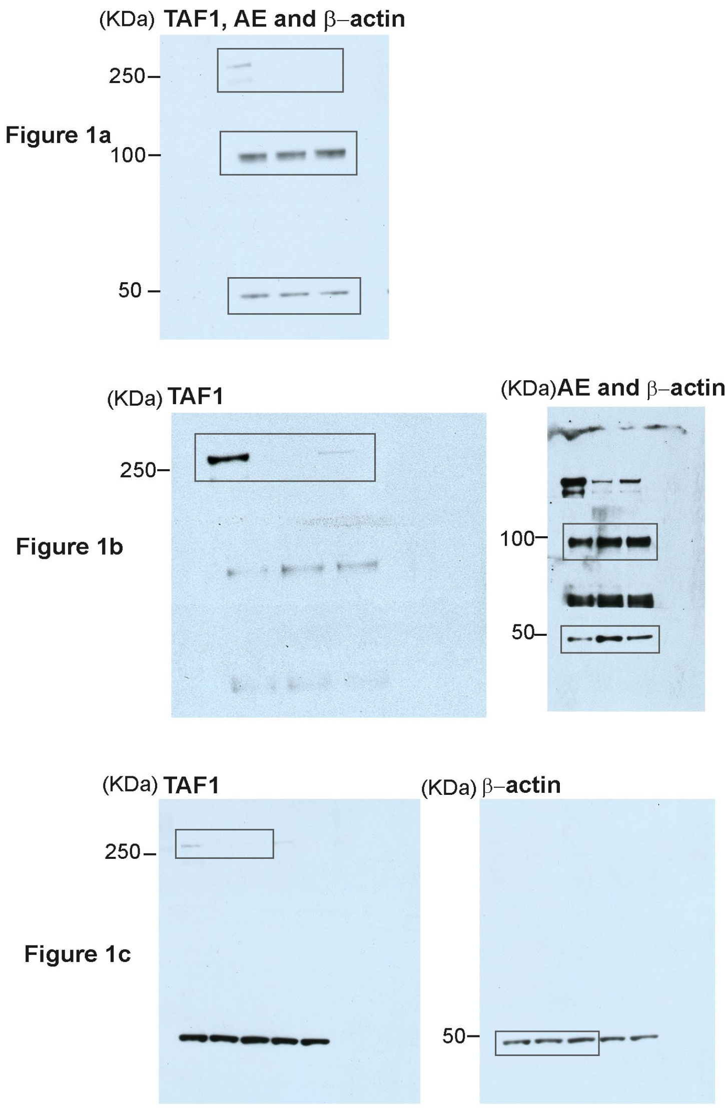

Supplementary Figure 5. Uncropped scans from figure 1. The boxes indicate the cropped images.

Figure 5a

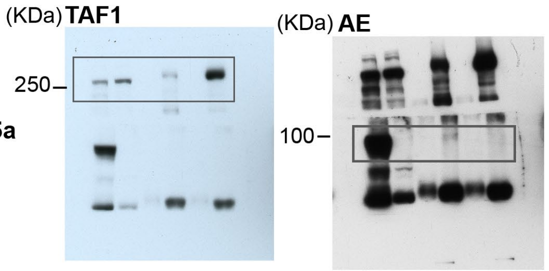

Figure 5b

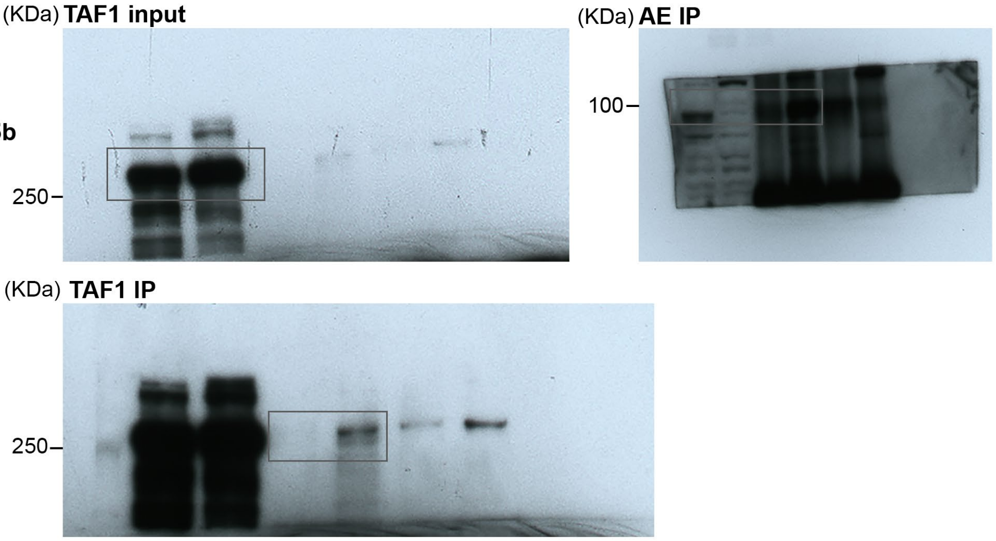

Figure 5d

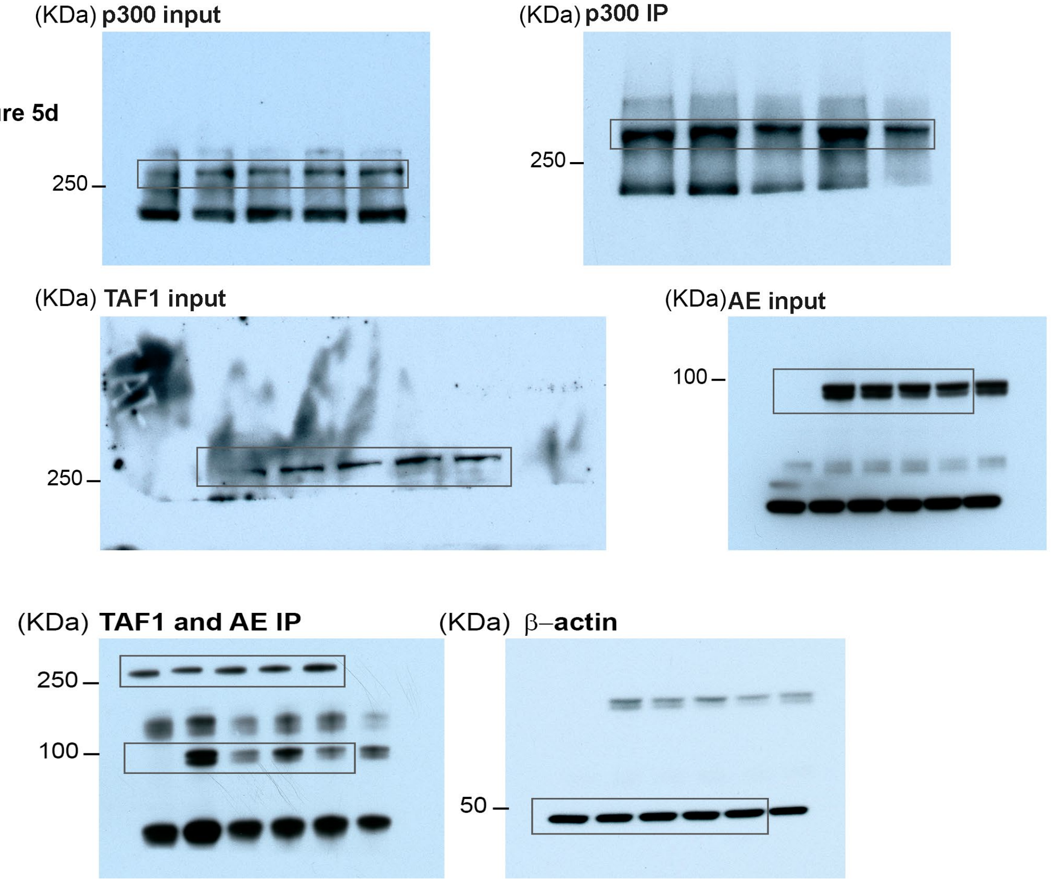

Figure 5e

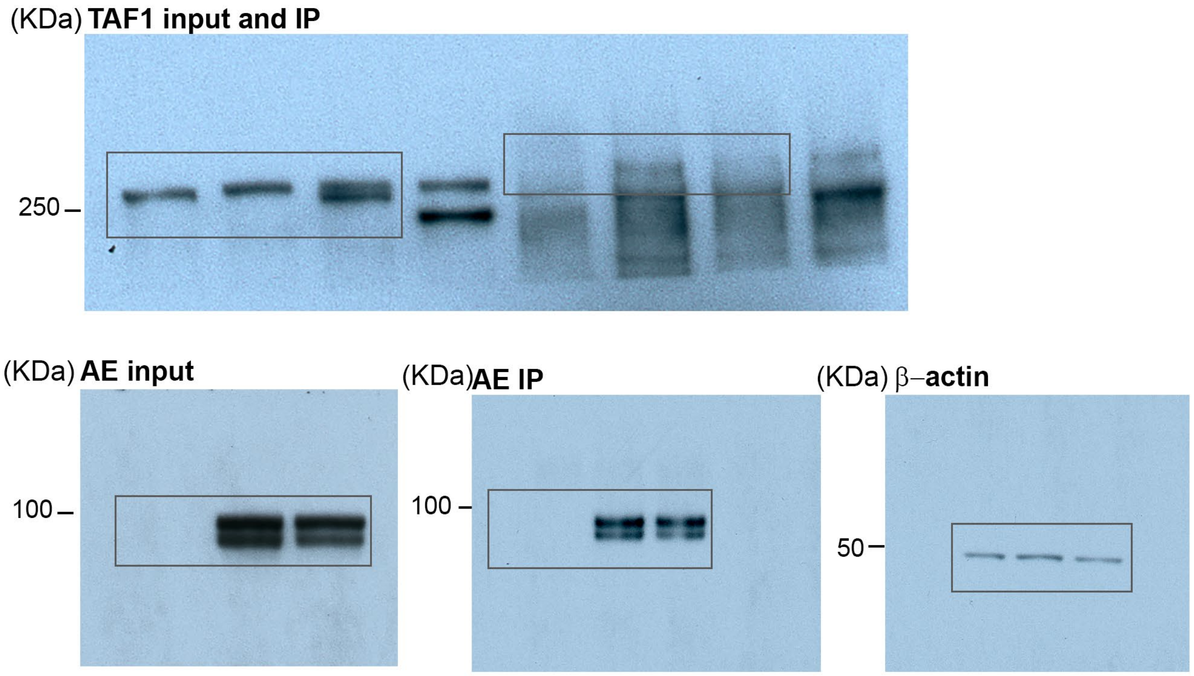

Supplementary Figure 6. Uncropped scans from figure 5. The boxes indicate the cropped images.

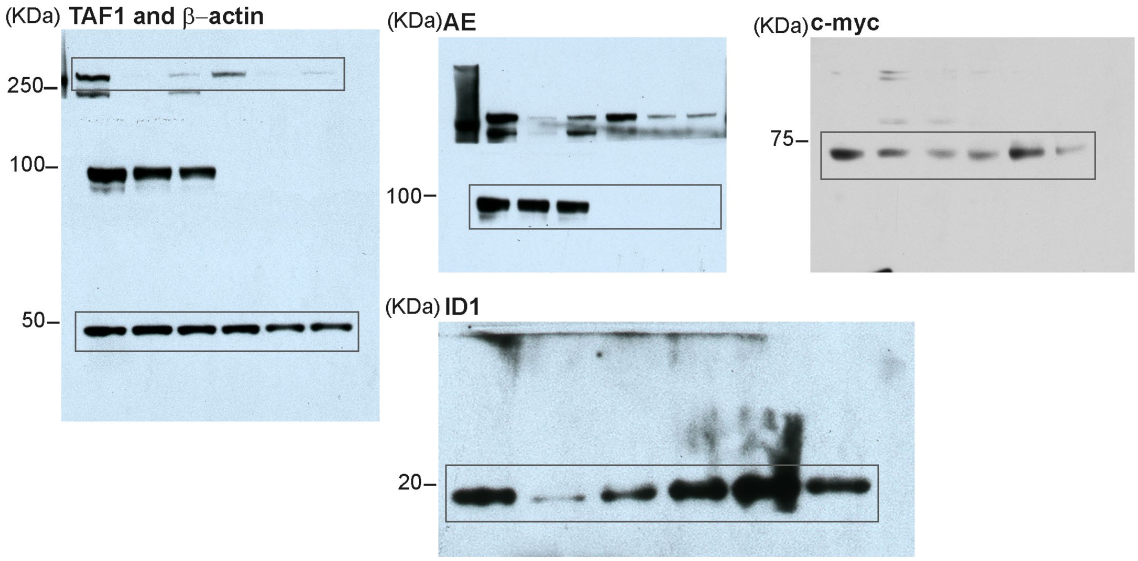

Supplementary Figure 7. Uncropped scans from figure 6d. The boxes indicate the cropped images.

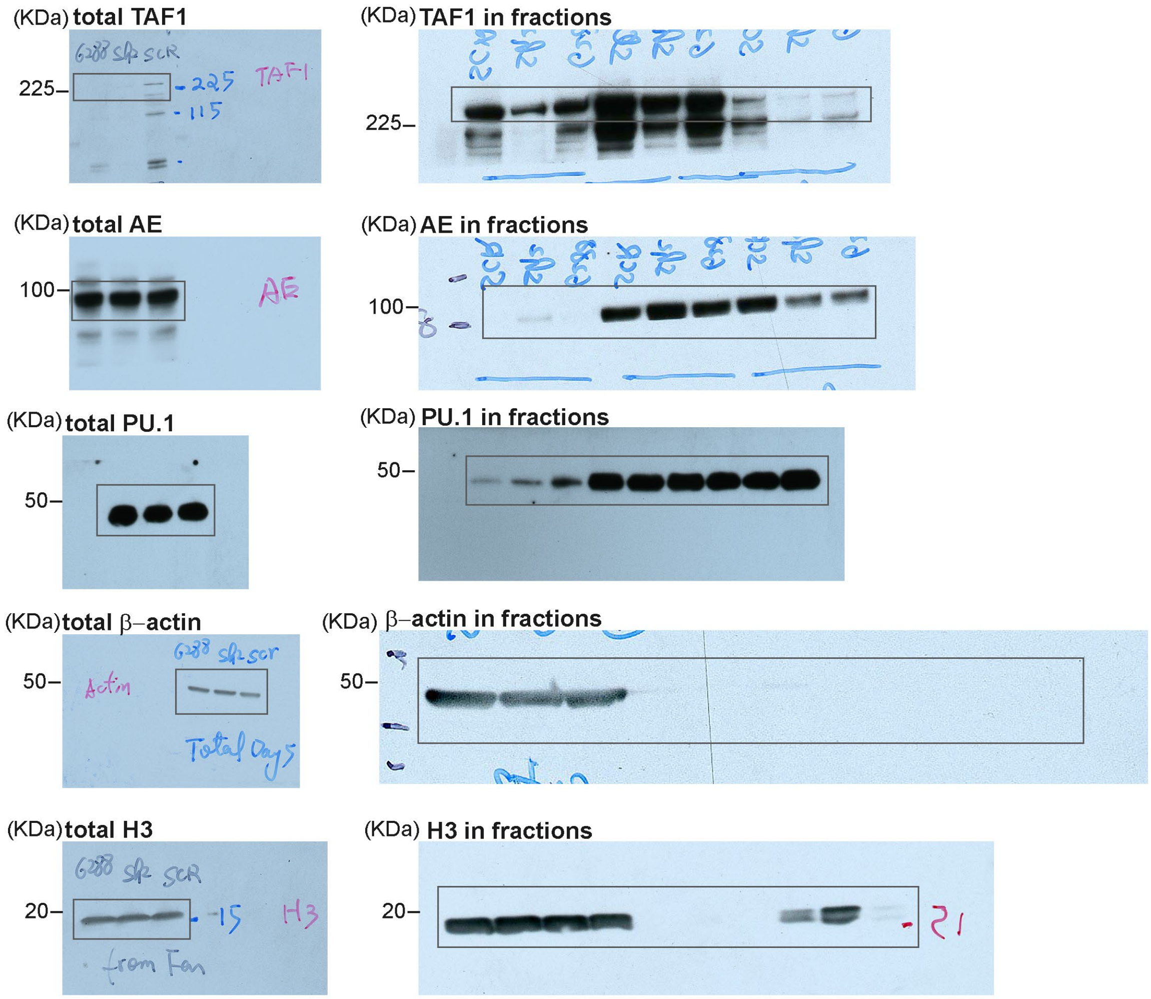

Supplementary Figure 8. Uncropped scans from figure 7a. The boxes indicate the cropped images.

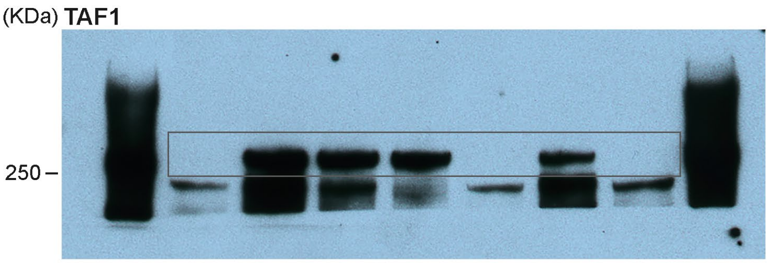

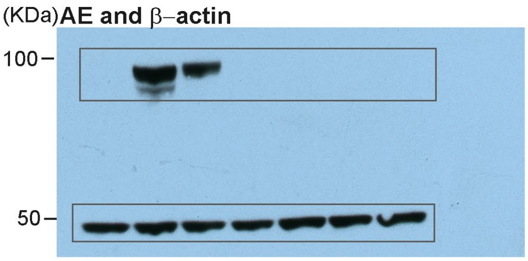

Supplementary Figure 9. Uncropped scans from supplementary figure 1a. The boxes indicate the cropped images.

Supplementary Table 1. Mass spectrometry analysis of proteins associated with AE.

unique	total	Reference	Gene symbol
12	30	Q06455_MTG8_HUMAN	RUNX1T1
13	19	Q01196_RUNX1_HUMAN	RUNX1
8	9	Q13951_PEBB_HUMAN	CBFB
2	2	P21675_TAF1_HUMAN	TAF1
2	2	Q92804_RBP56_HUMAN	TAF15

Supplementary Table 2. Antibodies used in the study.

Antibody (clone)	manufacturer	Catalog number	applications
TAF1	Santa Cruz	SC-735	ChIP-seq, IP, western (1:1000)
TAF1	Bethyl Laboratories	A303-505A	Western (1:1000)
TAF1	Millipore	04-1525	ChIP-seq, IP
TAF5	Bethyl Laboratories		Western (1:1000), IP
TAF6	Bethyl Laboratories	A301-275A	IP
TAF6	Bethyl Laboratories	A301-276A	Western(1:1000)
TAF7	Sigma	SAB1404438	Western, IP
TAF12	Proteintech	12353-1-AP	Western (1:1000), IP
TAF15	Abcam	Ab134916	Western (1:1000), IP
AML1-ETO	Invitrogen	PA5-40076	ChIP-seq
AML1-ETO	Diagenode	C15310197	ChIP-seq
ETO(C-20)	Santa Cruz	SC-9737	IP, western (1:1000)
AML1	Cell Signaling Technologies	4334s	Western (1:1000)
P300	Santa Cruz	SC-585	ChIP-seq, western (1:1000)
P300	Diagenode	C15200211	ChIP-seq
β-actin	Santa Cruz	SC-47778	Western (1:5000)
CARM1	Millipore	09818	Western (1:1000)
c-myc(9E10)	Santa Cruz	SC-40	Western (1:1000)
ID1 (C-20)	Santa Cruz	SC-488	Western(1:1000)
H3	Abcam	ab18521	Western (1:5000)
H3K4me1	Diagenode	C15200150	ChIP-seq
H3K27Ac	Millipore	07-360	ChIP-seq
Polymerase II (N-20)	Santa Cruz	SC-890X	ChIP-seq
FITC-Brdu	BD Pharmingen	559619	Brdu assay (1:100)
Human PE-CD11b	BD Pharmingen	555388	Flow cytometry (1:100)
Mouse perCP/cy5.5-Gr1 (RB6-8C5)	BD Pharmingen	108428	Flow cytometry (1:100)
Mouse PE-mac1 (m1/70)	BD Pharmingen	567397	Flow cytometry (1:100)
PE-Annexin V	BD Pharmingen	559763	Flow cytometry (1:20)
7-AAD	BD Pharmingen	559763	Flow cytometry (1:100)

Supplementary Table 3. Primers used in the study

Human *TAF1*	Forward: TTCCACACTCCAGTCAATGC
	Reverse: GTGTTTTGGCCCATTGTAGG
Human *18S*	Forward: CCCAGTAAGTGCGGGTCATA
	Reverse: GATCCGAGGGCCTCACTAAAC
Human *ID1*	Forward: CTACGACATGAACGGCTGTTACTC
	Reverse: CTTGCTCACCTTGCGGTTCT
AML1-ETO	Forward: AATCACAGTGGATGGGCCC
	Reverse: TGCGTCTTCACATCCACAGG
	Probe: CTGAGAAGCACTCCACAATGCCAGACT
Mouse *ID1*	Forward: CGACTACATCAGGGACCTGCA
	Reverse: GAACACATGCCGCCTCGG
Mouse *18S*	Forward: GTAACCCGTTGAACCCCATT
	Reverse: CCATCCAATCGGTAGTAGCG
Mouse *TAF1*	Forward: TGCTCGGAGAAAGAGGAAAA
	Reverse: GACTCCACAGGAGCCATCAT
Mouse *GAPDH*	Forward: CCTGACCTGCCGTCTAGAAAA
	Reverse: CTCCGACGCCTGCTTCAC

probe	source	identifier
Human *MYC*	Thermos Fisher	Hs00153408_m1
Human *ACTB*	Thermos Fisher	Hs01060665_g1
Human *CCND2*	Thermos Fisher	Hs00153380-m1
Human *CDKN2D*	Thermos Fisher	Hs01017690_g1
Human *CCND3*	Thermos Fisher	Hs00176481_m1
Mouse *ID1*	Thermos Fisher	Mm00775963_g1
Mouse *MYC*	Thermos Fisher	Mm00487804_m1
