# Supplementary material for: TAF1 plays a critical role in AML1-ETO driven leukemogenesis
Source: Nat Commun. 2019 Oct 29;10:4925. doi: 10.1038/s41467-019-12735-z (PMC6820555; doi:10.1038/s41467-019-12735-z)
Supplement: Supplementary file 3 — Reporting Summary [file 41467_2019_12735_MOESM3_ESM.pdf]

nature research

Corresponding author(s): Stephen Nimer

Reporting Summary

Nature Research wishes to improve the reproducibility of the work that we publish. This form provides structure for consistency and transparency in reporting. For further information on Nature Research policies, see Authors & Referees and the Editorial Policy Checklist.

Statistical parameters

When statistical analyses are reported, confirm that the following items are present in the relevant location (e.g. figure legend, table legend, main text, or Methods section).

n/a	Confirmed	
☐	☒	The exact sample size (*n*) for each experimental group/condition, given as a discrete number and unit of measurement
☐	☒	An indication of whether measurements were taken from distinct samples or whether the same sample was measured repeatedly
☐	☒	The statistical test(s) used AND whether they are one- or two-sided<br>*Only common tests should be described solely by name; describe more complex techniques in the Methods section.*
☐	☒	A description of all covariates tested
☐	☒	A description of any assumptions or corrections, such as tests of normality and adjustment for multiple comparisons
☐	☒	A full description of the statistics including central tendency (e.g. means) or other basic estimates (e.g. regression coefficient) AND variation (e.g. standard deviation) or associated estimates of uncertainty (e.g. confidence intervals)
☐	☒	For null hypothesis testing, the test statistic (e.g. *F*, *t*, *r*) with confidence intervals, effect sizes, degrees of freedom and *P* value noted<br>*Give P values as exact values whenever suitable.*
☒	☐	For Bayesian analysis, information on the choice of priors and Markov chain Monte Carlo settings
☒	☐	For hierarchical and complex designs, identification of the appropriate level for tests and full reporting of outcomes
☒	☐	Estimates of effect sizes (e.g. Cohen's *d*, Pearson's *r*), indicating how they were calculated
☐	☒	Clearly defined error bars<br>*State explicitly what error bars represent (e.g. SD, SE, CI)*

Our web collection on statistics for biologists may be useful.

Software and code

Policy information about availability of computer code

Data collection	Stemvison was used to scan the 6-well plates for colony formation in AE9a cells.
Data analysis	cutadapt v.1.15, kundajelab Aquas ChIPseq-Pipeline (git commit SHA1: 5e0e6f119b70c6e7a0a2578d38847f30e200df95), chipseeker v1.14.0, meme-chip v4.12.0, MACS2 v2.1.1.20160309, edaseq v.2.12.0., ENCODE3 atac-seq-pipeline (git commit: 2b693abd4550943be1e8d9a686a1050c1acab92c), deeptools v.3.1.1 . Statistical analysis was performed using Graphprism 6.0.

For manuscripts utilizing custom algorithms or software that are central to the research but not yet described in published literature, software must be made available to editors/reviewers upon request. We strongly encourage code deposition in a community repository (e.g. GitHub). See the Nature Research guidelines for submitting code & software for further information.

Data

Policy information about availability of data

All manuscripts must include a data availability statement. This statement should provide the following information, where applicable:

- Accession codes, unique identifiers, or web links for publicly available datasets
- A list of figures that have associated raw data
- A description of any restrictions on data availability

RNA-seq, ChIP-seq, and ATACserq data have been deposited in the Gene Expression Omnibus (GEO) under the accession codes GSE100446

Field-specific reporting

Please select the best fit for your research. If you are not sure, read the appropriate sections before making your selection.

☒ Life sciences ☐ Behavioural & social sciences ☐ Ecological, evolutionary & environmental sciences

For a reference copy of the document with all sections, see nature.com/authors/policies/ReportingSummary-flat.pdf

Life sciences study design

All studies must disclose on these points even when the disclosure is negative.

Sample size	No statistical mehod was used to predetermine thh sample size. Sample size was determined based on the literature.
Data exclusions	No data was excluded
Replication	Attempts at replication were successful.
Randomization	Mice in animal studies were assigned into each group randomly.
Blinding	Blind analysis was used for manual quantification of colony formation assays. In all other cases, blinding was not necessary because our analysis was objective.

Reporting for specific materials, systems and methods

Materials & experimental systems

n/a	Involved in the study
☐	☒ Unique biological materials
☐	☒ Antibodies
☐	☒ Eukaryotic cell lines
☒	☐ Palaeontology
☐	☒ Animals and other organisms
☒	☐ Human research participants

Methods

n/a	Involved in the study
☐	☒ ChIP-seq
☐	☒ Flow cytometry
☒	☐ MRI-based neuroimaging

Unique biological materials

Policy information about availability of materials

Obtaining unique materials	Unique materials are available from corresponding authors upon request.

Antibodies

Antibodies used	The antibodies used in the western blots: anti-TAF1 antibody (Santa Cruz, sc-735 or Bethlyl laboratories, Inc., A303-505A), anti-ETO antibody (c-20)(Santa Cruz, sc-9737), anti-β-actin antibody (Santa Cruz, sc-47778), anti-CARM1 antibody (Millipore, 09818), anti-c-myc antibody (clone 9E10, Santa Cruz, sc-40), anti-ID1 antibody (clone c-20, Santa Cruz, sc-488), anti-lamin B antibody (clone C-20, Santa Cruz, sc-6216), anti-H4 antibody (Cell Signaling Technologies, 2592s), anti-p300 antibody (N-15, Santa Cruz, sc-584), anti-AML1 antibody (Cell Signaling Technologies, 4334S), anti-TAF5 antibody (Bethlyl laboratories, Inc., A303-687A-M),

anti-TAF6 antibody (Bethlyl laboratories, Inc., A301-275A and A301-276A), anti-TAF12 antibody (Proteintech, 12353-1-AP), anti-TAF15 antibody (Abcam, ab134916), anti-TAF7 antibody (Sigma, SAB1404438);
The antibodies used in flow cytometry: anti-FITC-Brdu antibody (BD Pharmingen Brdu Flow Kit, 559619), anti-PE-human CD11b(mac1) (BD Pharmingen, 555388), anti-percp/cy5.5-mouse-Gr1 antibody (clone RB6-8C5, BD Pharmingen, 108428), anti-PE-mouse mac1 (clone m1/70, BD Pharmingen, 567397);

Validation: Antibodies were validated as noted by suppliers.

Eukaryotic cell lines

Policy information about cell lines

Cell line source(s)	Kasumi-1 and K562 were purchased from ATCC, SKNO-1 and OCI-AML3 were purchased from DSMZ.
Authentication	Cell lines were not externally authenticated.
Mycoplasma contamination	Mycoplasma contamination were routinely tested every month.
Commonly misidentified lines (See ICLAC register)	No misidentified cell lines were used in this study.

Animals and other organisms

Policy information about studies involving animals; ARRIVE guidelines recommended for reporting animal research

Laboratory animals	6-8 weeks female C57B1/6.SJL recipient mice were purchased from The Jackson Laboratory. All animal experiments were carried out according to the protocol approved by the IACUC of University of Miami.
Wild animals	The study didn't involve wild animals.
Field-collected samples	The study didn't involve samples collected from the field.

ChIP-seq

Data deposition

☒ Confirm that both raw and final processed data have been deposited in a public database such as GEO.

☒ Confirm that you have deposited or provided access to graph files (e.g. BED files) for the called peaks.

Data access links
May remain private before publication.

SuperSeries GSE100446

Files in database submission

Kasumi1-WT-H3K27ac_rep1.fc.signal.bw
Kasumi1-WT-H3K4me1_rep1.fc.signal.bw
Kasumi1-WT-ETO_rep1.fc.signal.bw
Kasumi1-WT-H3K27ac_rep2.fc.signal.bw
Kasumi1-WT-H3K4me1_rep2.fc.signal.bw
Kasumi1-WT-TAF1_rep3.fc.signal.bw
Kasumi1-WT-TAF1_rep4.fc.signal.bw
Kasumi1-WT-TAF1_rep5.fc.signal.bw
Kasumi1-WT-ETO_rep6.fc.signal.bw
Kasumi1-WT-H3K27ac.naive_overlap.filt.narrowPeak.gz
Kasumi1-WT-H3K4me1.naive_overlap.filt.narrowPeak.gz
Kasumi1-WT-TAF1_rep3.filt.narrowPeak.gz
Kasumi1-WT-TAF1_rep4.filt.narrowPeak.gz
Kasumi1-WT-TAF1_rep5.filt.narrowPeak.gz
Kasumi1-WT-ETO.filt.narrowPeak.gz
Kasumi1-WT-H3K27ac_rep1.fastq.gz
Kasumi1-WT-H3K4me1_rep1.fastq.gz
Kasumi1-WT-ETO_rep1.fastq.gz
Kasumi1-WT-input_rep1.fastq.gz
Kasumi1-WT-H3K27ac_rep2.fastq.gz
Kasumi1-WT-H3K4me1_rep2.fastq.gz
Kasumi1-WT-input_rep2.fastq.gz
Kasumi1-WT-TAF1_rep3.fastq.gz
Kasumi1-WT-input_rep3.fastq.gz
Kasumi1-WT-TAF1_rep4.fastq.gz
Kasumi1-WT-input_rep4.fastq.gz
Kasumi1-WT-TAF1_rep5.fastq.gz
Kasumi1-WT-input_rep5.fastq.gz
Kasumi1-WT-ETO_rep6.fastq.gz

Kasumi1-WT-input_rep6.fastq.gz
Kasumi1-WT-input_rep11.fastq.gz
Kasumi1-WT-P300_rep2.fastq.gz
Kasumi1-WT-P300_rep2.fc.signal.bw
Kasumi1-WT-P300_rep11.fastq.gz
Kasumi1-WT-P300_rep11.fc.signal.bw
Kasumi1-WT-P300.filt.narrowPeak.gz
Kasumi_shCtr_AE_rep1_peak.txt
Kasumi_shCtr_AE_rep1.bw
Kasumi_shCtr_AE_rep1.fastq.gz
Kasumi_shCtr_AE_rep2_peak.txt
Kasumi_shCtr_AE_rep2.bw
Kasumi_shCtr_AE_rep2.fastq.gz
Kasumi_shTAF1_AE_rep1_peak.txt
Kasumi_shTAF1_AE_rep1.bw
Kasumi_shTAF1_AE_rep1.fastq.gz
Kasumi_shTAF1_AE_rep2_peak.txt
Kasumi_shTAF1_AE_rep2.bw
Kasumi_shTAF1_AE_rep2.fastq.gz

Genome browser session
(e.g. UCSC)

Provide a link to an anonymized genome browser session for "Initial submission" and "Revised version" documents only, to enable peer review. Write "no longer applicable" for "Final submission" documents.

Methodology

Replicates

For histones, we used naïve overlap for peak file. For peaks with two replicates, we used optimal replicate IDR < 0.05 for peak calls. For WT TAF1, where we had three replicates, we used optimal pseudo-replicate IDR < 0.05 for internal peaks and considered TAF1 peaks where a given sample overlapped other samples.

Sequencing depth

Kasumi1-WT-H3K27ac_rep1, total: 67.4M, unique mapped: 42.2M, length: 76 , single-end
Kasumi1-WT-H3K4me1_rep1, total: 53.4M, unique mapped: 43.9M, length: 76 , single-end
Kasumi1-WT-ETO_rep1, total: 50.5M, unique mapped: 36.7M, length: 76 , single-end
Kasumi1-WT-input_rep1, total: 51.4M, unique mapped: 42.0M, length: 76 , single-end
Kasumi1-WT-H3K27ac_rep2, total: 31.7M, unique mapped: 24.3M, length: 76 , single-end
Kasumi1-WT-H3K4me1_rep2, total: 29.1M , unique mapped: 21.8M, length: 76 , single-end
Kasumi1-WT-input_rep2, total: 23.9M , unique mapped: 19.2, length: 76 , single-end
Kasumi1-WT-TAF1_rep3, total: 32.7M, unique mapped: 3.6M, length: 76 , single-end
Kasumi1-WT-input_rep3, total: 43.4M, unique mapped: 34.3M, length: 76 , single-end
Kasumi1-WT-TAF1_rep4, total: 36.9M , unique mapped: 24.3M, length: 76 , single-end
Kasumi1-WT-input_rep4, total: 35.6M , unique mapped: 27.7M, length: 76 , single-end
Kasumi1-WT-TAF1_rep5, total: 23.0M, unique mapped: 12.6M, length: 76 , single-end
Kasumi1-WT-input_rep5, total: 31.4M, unique mapped: 22.0M, length: 76 , single-end
Kasumi1-WT-ETO_rep6, total: 68.2M, unique mapped: 29.1M, length: 76 , single-end
Kasumi1-WT-input_rep6, total: 38.0M, unique mapped: 4.9M, length: 76 , single-end
Kasumi1-WT-P300_rep2, total:27.0M mapped:26.0M length:76 single-end
Kasumi1-WT-P300_rep11, total:55.0M mapped:52.2M length:36 single-end
Kasumi1-WT-input_rep11, total:6.3M mapped:4.3M length:36 single-end
Kasumi-shCtrl_AE_rep1, total: 16.6M mapped: 12.5M length: 73, single-end
Kasumi-shCtrl_AE_rep2, total: 11.9M mapped: 8.9M length: 73, single-end
Kasumi-shTAF1_AE_rep1, total: 17.1M mapped: 12.9M length: 73, single-end
Kasumi-shTAF1_AE_rep2, total: 15.7M mapped: 11.5M length: 73, single-end

Antibodies

The antibodies used in ChIP-seq: anti-TAF1 antibody (Santa Cruz, sc-735; Millipore, 04-1525) anti-AML1-ETO antibody (Diagenode, C15310197), H3K4me1 (C15200150) and H3K27Ac (Millipore 07-360), anti-p300 antibody (Santa Cruz, sc-585 and Diagenode C15200211), polymerase II (Santa Cruz, sc-899X).

Peak calling parameters

Processed with Kundaje lab ChIPseq Pipeline (git commit SHA1: 5e0e6f119b70c6e7a0a2578d38847f30e200df95) (https://github.com/kundajelab/chipseq_pipeline) with -peak_caller macs2 -species hg38

Data quality

Performed Fastqc and fastq_screen to verify quality and lack of contamination

Software

Base calling using Illumina RTA 2.4.11
Trimmed fastq files with cutadapt v.1.15 --nextseq-trim=20 -u 2 -u -2 -m 18
Processed with Kundaje lab Aquas Pipeline (git commit SHA1: 5e0e6f119b70c6e7a0a2578d38847f30e200df95) with -peak_caller macs2 -species hg38

Flow Cytometry

Plots

Confirm that:

☒ The axis labels state the marker and fluorochrome used (e.g. CD4-FITC).

☒ The axis scales are clearly visible. Include numbers along axes only for bottom left plot of group (a 'group' is an analysis of identical markers).

☒ All plots are contour plots with outliers or pseudocolor plots.

☒ A numerical value for number of cells or percentage (with statistics) is provided.

Methodology

Sample preparation	Kasumi-1 cells and K562 cells were transduced with scrambled shRNA or TAF1 shRNA viruses for days, cells were washed with PBS and stained with individual antibodies.<br>Purified CD34+ cells were transduced with scrambled shRNA or TAF1 directed shRNA followed by puromycin selection for 48 hours. After puromycin selection, GFP tagged Migr1 or Migr-AE retroviruses were introduced into CD34+ cells and the GFP+ CD34+ cells were sorted.<br>For myeloid differentiation, CD34+ were growned in myeloid differentiation medium for 4 days and then stained with human mac1 antibody.<br>Bone marrow cells were collected from Mx1-cre or AE knockin mice. After ACK (Ammonium-Chloride-Potassium) lysis, bone marrow cells were cultured in vitro for 4 days. Mouse Mac1 and Gr1 antibodies were used to stain myeloid differentiated cells.
Instrument	BD FACS. Canto-II was used for analysis and BD FACS Aria-II sorter was used for sorting.
Software	FlowJo_V10 was used to analyze the data.
Cell population abundance	For GFP positive-sorted cells, the purity was determined by the percentage of GFP cells during sorting.
Gating strategy	The gating strategy was initially FSC-A by SSC-A, followed by FSC-A by FSC-W and then followed by SSC-A by SSC-W. For each color, we gated based on self-prepared compensation control.

☒ Tick this box to confirm that a figure exemplifying the gating strategy is provided in the Supplementary Information.
